# Solvated Ion Intercalation in Graphite: Sodium and Beyond

**DOI:** 10.3389/fchem.2020.00432

**Published:** 2020-05-21

**Authors:** Jooha Park, Zheng-Long Xu, Kisuk Kang

**Affiliations:** ^1^Department of Materials Science and Engineering, Research Institute of Advanced Materials (RIAM), Seoul National University, Seoul, South Korea; ^2^Department of Industrial and Systems Engineering, The Hong Kong Polytechnic University, Kowloon, Hong Kong; ^3^Institute of Engineering Research, College of Engineering, Seoul National University, Seoul, South Korea; ^4^Center for Nanoparticle Research, Institute of Basic Science, Seoul National University, Seoul, South Korea

**Keywords:** graphite, co-intercalation, anode materials, Li-ion batteries, Na-ion batteries, K-ion batteries, Mg-ion batteries, Ca-ion batteries

## Abstract

Reversible intercalation of guest ions in graphite is the key feature utilized in modern battery technology. In particular, the capability of Li-ion insertion into graphite enabled the successful launch of commercial Li-ion batteries 30 years ago. On the road to explore graphite as a universal anode for post Li-ion batteries, the conventional intercalation chemistry is being revisited, and recent findings indicate that an alternative intercalation chemistry involving the insertion of solvated ions, designated as co-intercalation, could overcome some of the obstacles presented by the conventional intercalation of graphite. As an example, the intercalation of Na ions into graphite for Na-ion batteries has been perceived as being thermodynamically impossible; however, recent work has revealed that a large amount of Na ions can be reversibly inserted in graphite through solvated-Na-ion co-intercalation reactions. More recently, it has been extensively demonstrated that with appropriate electrolyte selection, not only Na ions but also other ions such as Li, K, Mg, and Ca ions can be co-intercalated into a graphite electrode, resulting in high capacities and power capabilities. The co-intercalation reaction shares a lot in common with the conventional intercalation chemistry but also differs in many respects, which has attracted tremendous research efforts in terms of both fundamentals and practical applications. Herein, we aim to review the progress made in understanding the solvated-ion intercalation mechanisms in graphite and to comprehensively summarize the state-of-the-art achievements by surveying the correlations among the guest ions, co-intercalation conditions, and electrochemical performance of batteries. In addition, the advantages and challenges related to the practical application of graphite undergoing co-intercalation reactions are presented.

## Introduction

The ever-growing energy demands associated with global economic growth combined with the current dependency on unsustainable energy resources and related environmental concerns has motivated our modern society to explore green and sustainable energy resources such as solar and wind. These naturally intermittent energy resources require the development of reliable energy storage systems (ESSs), and rechargeable batteries are among the most promising candidates. Since the early 1990s, lithium-ion batteries (LIBs) have dominated the energy storage market with their high energy densities and reliable battery performance (Kang et al., 2006; Armand and Tarascon, 2008; Dunn et al., 2011). However, the surging global market for electric vehicles has raised concerns about the sustainable supply of LIBs, particularly the uneven distribution of lithium resources in the world, leading to demand for alternative battery chemistries (Kim et al., 2012; Choi and Aurbach, 2016; Ponrouch et al., 2016; Olivetti et al., 2017). In this respect, increasing attention has been focused on post LIBs, including Sodium-ion batteries (SIBs) (Pan et al., 2013; Xiang et al., 2015; Kim et al., 2016a; Kim J. et al., 2018; Laziz et al., 2018; Lee et al., 2018), Magnesium-ion batteries (MIBs) (Muldoon et al., 2014), and Calcium-ion batteries (CIBs) (Gummow et al., 2018; Ponrouch and Palacin, 2018; Wang et al., 2018) as alternative-ion battery technologies, considering the similar physicochemical properties of lithium and alkali metal (i.e., Na, K)/alkaline earth metal (i.e., Mg, Ca) elements.

**Figure 1:**
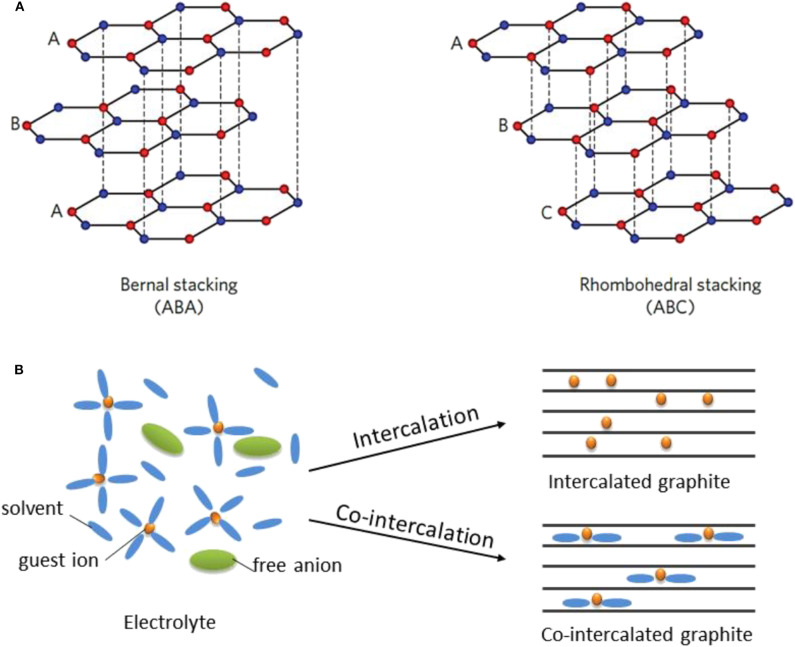
**(A)** Schematic illustration of graphite structure with two stacking sequences including ABAB (2H) and ABCABC (3R). ABAB stacking is known to be the main type of stacking for the graphite. **(B)** Schematic diagrams of the coordination structure in electrolyte, the intercalated graphite and the co-intercalated graphite, respectively. Co-intercalation involves the insertion of solvated ions. **(A)** Reproduced from Li et al. (2019) with permission from the Royal Society of Chemistry.

Graphite has been the standard anode material for LIBs since the 1990s. For post-LIBs based on intercalation chemistry, graphite is also regarded as a preferred anode because in addition to the merits of graphite itself (including its low cost and chemical/electrochemical stability), one can learn from past lessons on the electrode design of LIBs and/or employ similar battery manufacturing lines as those used for LIBs with high industrial convenience (Xu et al., 2018b; Li et al., 2019). Graphite consists of honeycomb carbon layers weakly bound by van der Waals interaction, as depicted in Figure 1A, with hexagonal ABA or rhombohedral ABC stacking and an interlayer distance of ≈0.335 nm (Li et al., 2019). Guest ions can intercalate into the graphite galleries by forming graphite intercalation compounds (GICs). The history of GICs can be traced back to 1840s when Schaffautl et al. attempted to intercalate H_2_SO_4_ into graphite (Schafhaeutl, 1840). Since then, various reagents have been intercalated into graphite for applications ranging from superconducting materials and catalysts to hydrogen storage materials and battery electrodes (Besenhard et al., 1980; Dresselhaus and Dresselhaus, 1981). The intercalation of Li ions into graphite in non-aqueous electrochemical cells was achieved in the 1990s (Fong et al., 1990; Shu et al., 1993; Dahn et al., 1995), laying the foundation for modern LIBs. As illustrated in Figure 1B, for typical Li intercalation in graphite, the solvated Li ions in the electrolyte are de-solvated, and bare Li ions are subsequently inserted, yielding a theoretical lithium storage capacity of ≈372 mAh g^−1^ at a potential of ≈0.15 V vs. Li^+^/Li.

Despite the unique capability of graphite to host various intercalants including Li, K, Cs, and Rb, the amount of Na that can be reversibly intercalated into graphite is known to be unexpectedly small (≈NaC_186_) (Doeff et al., 1993). Similar results have also been reported for MIBs (Liu et al., 2016) and CIBs (Takeuchi et al., 2011). Theoretical studies have revealed that the strong local interaction between Na (or Mg, Ca) ions and graphene layers dominantly destabilizes the GICs (Liu et al., 2016; Yoon et al., 2017), consequently leading to low Na (or Mg, Ca)-ion storage capacities in graphite anodes. However, our group (Kim et al., 2015a) and (Jache and Adelhelm, 2014) independently observed that Na can be reversibly stored in graphite in a large quantity through co-intercalation reactions, where solvated Na ions are intercalated together into graphite galleries, forming a ternary GIC (*t*-GIC) (Figure 1B). Motivated by these findigs (Kim et al., 2015b; Seidl et al., 2017), the co-intercalation of other ions (i.e., Li^+^, K^+^, Mg^2+^, Ca^2+^) in graphite (Kim et al., 2016b, 2017; Kim D. M. et al., 2018; Park et al., 2019; Prabakar et al., 2019) and the electrochemical performance of co-intercalated graphite (Cohn et al., 2016a; Hasa et al., 2016) have been extensively investigated in recent years.

In this review, we undertook a careful survey and analysis of the state-of-the-art knowledge on the co-intercalation behaviors of alkali (Li, Na and K) and alkaline earth (Mg, Ca) metal ions into graphite. Recent experimental and theoretical work will be overviewed and issues related to the differences between intercalation and co-intercalation will be discussed. Advantages and challenges of co-intercalation reactions are presented as guidelines for the design of graphite anodes exploiting co-intercalation. Finally, we will discuss how the major challenges can be potentially resolved to enable the practical application of co-intercalation graphite anodes.

## Solvated Alkali and Alkaline Earth Metal-ion Intercalation in Graphite

### Na Co-intercalation

**Figure 2:**
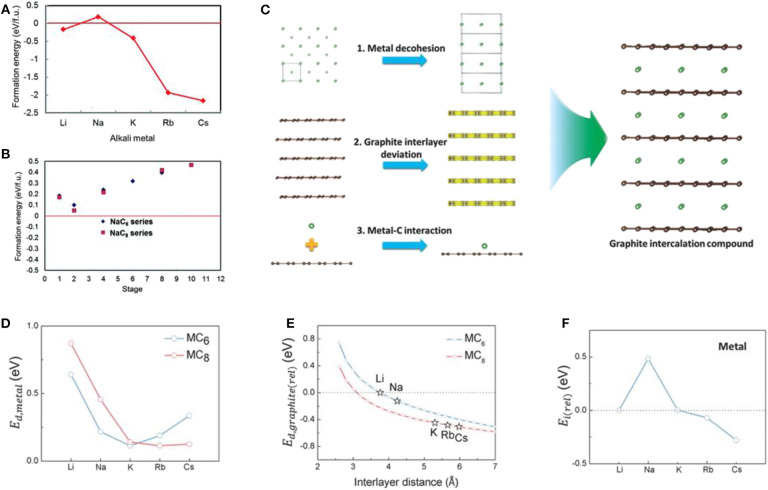
**(A)** Formation energies of AMC_6_ structured GICs (AM: Li, Na, K, Rb, and Cs). **(B)** Formation energies of NaC_6_ and NaC_8_ GICs with different stage number. **(C)** Schematic images of three different contributing factors to the formation energy of AM-GICs; Energy factors from **(D)** metal decohesion, **(E)** AM intercalation and interlayer space, and **(F)** interaction between AM and graphene single layer. **(A,B)** Reproduced from Moriwake et al. (2017) with permission from the Royal Society of Chemistry. **(C–F)** Reproduced from Yoon et al. (2017) with permission from Wiley-VCH.

A low-stage Na intercalated binary graphite intercalation compound (*b*-GIC) has never been experimentally observed, unlike other alkali-based binary GICs such as LiC_6_ and KC_8_ (Metrot et al., 1979; Ge and Fouletier, 1988; Adhoum et al., 2006; Moriwake et al., 2017). Because of the incapability of Na insertion in graphite, most previous efforts in the development of Na storage anodes have focused on other types of carbon and its derivatives (Doeff et al., 1993; Stevens and Dahn, 2001; Irisarri et al., 2015; Yun et al., 2015; Kim and Kim, 2018; Xu et al., 2018b). A few theoretical papers have focused on the origin of the thermodynamic instability of Na *b*-GICs (Figures 2A,B) (Nobuhara et al., 2013; Okamoto, 2014; Yoon et al., 2017). Wang et al. and Liu et al. rationalized the cause of the abnormal instability of Na insertion by deconvoluting the formation energies of GICs into three potential reactions (Wang et al., 2014; Liu et al., 2016): (1) the reconstruction of graphite, (2) metal intercalation, and (3) other remaining energies. Although the instability of Na *b*-GICs was found to be mainly related to the third reaction, a detailed investigation of those remaining energies was not explored. More recently, Yoon et al. elucidated the main factors contributing to the third reaction (Yoon et al., 2017). Density functional theory (DFT) calculations on the alkali metal-GICs (alkali metal: Li, Na, K, Rb, and Cs) were performed to investigate the thermodynamic instability of the stage 1 alkali metal-GICs. As indicated in Figure 2C, three primary changes occurring during the formation of GICs were scrutinized: (i) the alkali metal decohesion from pristine bcc-type structures, (ii) the structural deviation of graphite, and (iii) the local interaction between the single layer of graphite and the alkali metal ion. The computational results indicated that the energy of (iii) was the determining factor and linearly followed the trend of the formation energies of alkali metal-GICs, as depicted in Figure 2F, with the abnormality displayed only in the Na case. In contrast, trends for the energies related to (i) and (ii) were not observed peculiarly for Na GICs (Figures 2D,E, respectively). This finding suggests that the instability of the Na GIC framework can be mainly attributed to the elemental instability of Na adsorption on the graphene layer, which induces unfavorable local interaction between Na ions and graphite.

**Figure 3:**
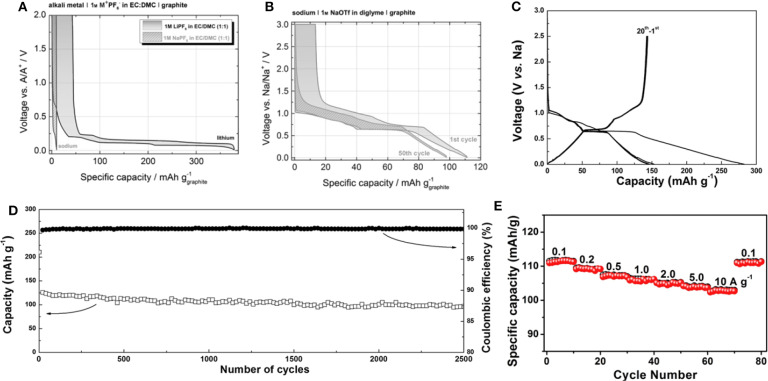
Charge and discharge profiles of Li/Na-based graphite cells with **(A)** conventional carbonate-based lithium electrolyte and **(B)** ether-based sodium electrolyte. **(C)** Charge and discharge profiles of natural graphite with Na-based DEGDME electrolyte. **(D)** Cycle stability, and **(E)** rate capability of Na ether co-intercalation reaction. **(A,B)** Reproduced from Jache and Adelhelm (2014) with permission from Wiley-VCH. **(C,D)** Reproduced from Kim et al. (2015a) with permission from Wiley-VCH. **(E)** Reproduced from Zhu et al. (2015) with permission from Elsevier.

Jache et al. and Kim et al. suggested a new approach to utilize graphite as an anode for Na-ion batteries using solvent co-intercalation chemistry (Jache and Adelhelm, 2014; Kim et al., 2015a). Jache et al. showed that the use of a graphite electrode in an electrochemical cell with sodium triflate (NaOTf) in diglyme electrolyte enabled Na-ion insertion, with a reversible specific capacity of ≈100 mAh g^−1^ and a coulombic efficiency of ≈99.87%; in contrast, a reference cell with NaPF_6_ in ethylene carbonate/diethyl carbonate (EC/DEC) electrolyte exhibited negligible capacity, as shown in Figures 3A,B (Jache and Adelhelm, 2014). The authors assumed that the composition of the fully sodiated GIC was Na(diglyme)_2_C_20_ based on the capacity; however, an explanation for the ratio between Na and diglyme molecules in the GIC was not provided. Kim et al. also demonstrated that a graphite electrode delivered a reversible capacity of ≈150 mAh g^−1^ in a cell employing DEGDME electrolyte (Figure 3C) (Kim et al., 2015a). Moreover, surprisingly, the graphite electrode stably delivered capacity over 2,500 cycles at a current density of 500 mA g^−1^, as shown in Figure 3D. *Ex-situ* X-ray photoelectron spectroscopy (XPS) and Fourier-transform infrared (FTIR) spectroscopy analysis confirmed that Na ions were co-intercalated with ether molecules into the graphite galleries. By monitoring the weight change of the graphite electrode during discharge and charge, the authors demonstrated that the Na:diglyme ratio in the *t*-GIC was actually 1:1 (Kim et al., 2015b). This ratio was further supported by energy-dispersive X-ray (EDX) spectroscopy analysis, which revealed the presence of 3 oxygens per Na in *t*-GIC with diglyme electrolytes. This finding indicated that one diglyme is coordinated with one Na ion in the co-intercalated graphite, suggesting that the composition of sodiated graphite is Na(digylme)C_21_. In addition to the excellent reversibility of graphite, the unexpectedly high rate capability of the co-intercalation has been also demonstrated. Upon increasing the current density from 0.1 to 10 A g^−1^, 92% of the capacity observed at 0.1 A g^−1^ was stably retained (Figure 3E) (Zhu et al., 2015).

**Figure 4:**
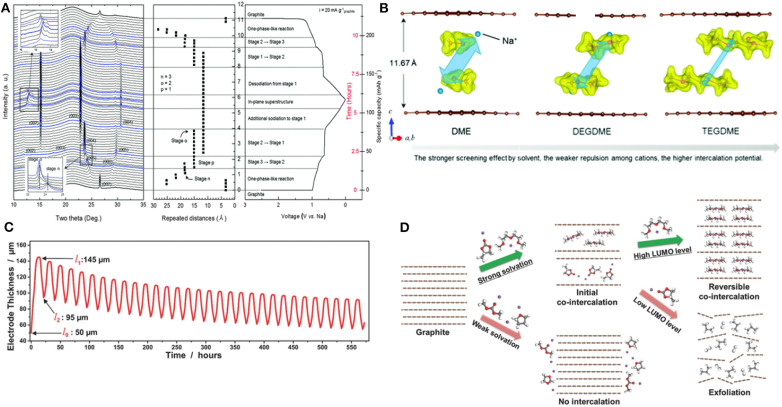
**(A)** *In operando* XRD patterns of graphite during electrochemical cycling in SIBs. **(B)** Possible configurations of ether-Na complex intercalated graphite compounds with different ether solvents. **(C)** Change of electrode thickness during cycling of graphite with ether-based electrolyte in SIBs. **(D)** Schematic illustration of the conditions for reversible Na co-intercalation. **(A,B)** Reproduced from Kim et al. (2015b) with permission from the Royal Society of Chemistry. **(C)** Reproduced from Goktas et al. (2018b) with permission from Wiley-VCH. **(D)** Reproduced from Yoon et al. (2017) with permission from Wiley-VCH.

An electrochemical mass spectroscopy study showed that the electrolyte decomposition only partly occurs in the first cycle but is restricted in further cycles (Goktas et al., 2018b). With the absence of the notable signature of solid electrolyte interphase (SEI) layers or broken fractions in transmission electron microscopy (TEM) images, it was speculated that even if SEI layers exist and break in the first cycle because of the large volume change originating from solvent co-intercalation, the new SEI layers did not form in further cycles. This SEI-free nature was believed to contribute to enhancing the charge-transfer kinetics in the process of Na intercalation and deintercalation (Goktas et al., 2018b; Jow et al., 2018). To better understand the Na storage mechanism in graphite, the structural evolution of graphite during Na de/intercalation was probed using synchrotron *in operando* X-ray diffraction (XRD) (Kim et al., 2015b). According to the XRD patterns presented in Figure 4A, the pristine graphite structure transformed into multiple new phases during the de/intercalation process, and the initial structure was recovered after cycling. The evolution of the XRD patterns clearly demonstrated that the typical staging behaviors of graphite occurred during co-intercalation. During the initial sodiation steps, the graphite electrode underwent one-phase-like reactions, which involved many different staging structures changing sensitively with small variations of Na storage contents. The subsequent phase transformations continued by forming stage 3, stage 2, and stage 1 with further sodium-complex intercalations. During the desodiation steps, the reverse phase transformations were observed, indicating the reversibility of the Na de/intercalation, which was consistent with the *ex-situ* XRD analysis (Zhu et al., 2015). The *c*-lattice parameter was shown to systematically change by ≈3.4 Å for each staging process, with that of stage 1 being as large as 11.62 Å. On the basis of the DFT calculations and XRD analysis, Kim et al. proposed a detailed Na-GIC structure and reported that the structure with double stacking of the [Na-DEGDME]^+^ complex located at one-third and two-third of the height of the graphite galleries was the most energetically stable and best fit with the XRD data (Figure 4B). In addition, a dilatometry experiment conducted by Goktas et al. revealed that despite the large *c*-lattice variation in *t*-GIC structures, the practical volume change is approximately 70%−100% on the electrode scale (Figure 4C) (Goktas et al., 2018b).

The co-intercalation reaction strongly depends on the selection of solvents in the electrolytes because the coordination structures of Na ions are determined by the nature of the co-intercalating solvent molecules (Kim et al., 2015a; Jache et al., 2016; Goktas et al., 2018a; Xu et al., 2019). Indeed, the most favorable coordination number of one alkali metal ion in glyme-based electrolytes ranges from 4 to 7, leading to complexes with different structures (Matsui and Takeyama, 1998; Rhodes et al., 2002; Henderson, 2006; Kim et al., 2016b). Xu et al. demonstrated that the Na storage potentials increase with the chain length of the ether solvent, varying from 0.59 to 0.65 to 0.77 V (vs. Na/Na^+^) in electrochemical cells employing dimethoxyethane (DME), DEGDME, and tetraethylene glycol dimethyl ether (TEGDME), respectively (Xu et al., 2019). This variation was attributed to the solvent molecules with longer chains providing more efficient screening against the unfavorable interaction between the Na ions and host. Yoon et al. scrutinized the role of solvent molecules with respect to the thermodynamic stability of ternary Na GICs (Yoon et al., 2017). A Na-DEGDME complex intercalated GIC was shown to have a greatly reduced formation energy of −0.87 eV compared with that of NaC_6_ conformation (0.03 eV) due to the effective screening of the interaction between Na and graphene, promoting the generation of stable co-intercalated GICs. Furthermore, the authors proposed two conditions for solvent selection for reversible Na storage in graphite, as illustrated in Figure 4D: (i) a large solvation energy and (ii) high lowest unoccupied molecular orbital (LUMO) levels of the [Na–solvent]^+^ complexes. A strong solvation capability of the solvent to Na ions enhances the stability of [Na–solvent]^+^ complexes during the intercalation. For example, the presence of multiple oxygen atoms in ether-based solvents results in the strong solvation structure of Na ions such as in DME, DEGDME, and TEGDME, which should be maintained during the co-intercalation process. For the second condition, if the LUMO level of the [Na–solvent]^+^ complexes is not sufficiently high or if it is even lower than the Fermi level of graphite, an energetically downhill reaction will inevitably occur, leading to electrolyte decomposition and gas evolution, or exfoliation eventually. Thus, the LUMO level of the solvent in the Na-ion complex should be reasonably higher than that of the Fermi level of graphite. This also explains why the intercalation of [Li–propylene carbonate (PC)]^+^ complexes, whose LUMO level is located below the Fermi level of graphite, involve significant exfoliation of graphite structures, as observed experimentally (Chung et al., 2000).

### Li and K Co-intercalation

**Figure 5:**
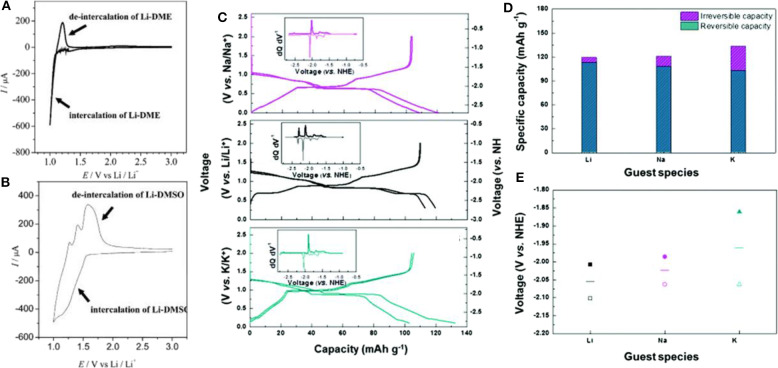
Cyclic voltammetry profiles of graphite cells with **(A)** DME-based Li electrolyte, and **(B)** DMSO-based Li electrolyte. **(C)** Analogous charge and discharge profiles of graphite with Na, Li, and K co-intercalation reactions. **(D)** Specific capacities and **(E)** average voltages of Li, Na and K co-intercalation reactions (filled symbols: charge voltage, empty symbols: discharge voltage, lines: average voltage) **(A,B)** Reproduced from Abe et al. (2004) with permission from the Electrochemical Society. **(C–E)** Reproduced from Kim et al. (2016b) with permission from the Royal Society of Chemistry.

Co-intercalation reactions of Li ions and solvent molecules in graphite were reported prior to Na co-intercalation reactions (Abe et al., 2002, 2004). In the development of LIBs, co-intercalation was regarded as undesirable because it usually led to exfoliation of graphite and gas evolution reactions, resulting in failure of the cell (Wagner et al., 2005). In 2004, Abe et al. reported the first reversible solvated-Li-ion co-intercalation reaction in graphite (Abe et al., 2004). When they employed solvents with high donor number in the electrolyte, partly reversible co-intercalation was observed to occur in DME and dimethyl sulfoxide (DMSO), unlike the co-intercalation in PC case (Figures 5A,B, respectively). The characteristic cathodic and anodic peaks differed from those of the conventional bare Li intercalation or solvent decomposition reaction. In addition, the presence of low-angle XRD peaks for the discharged graphite was considered additional experimental evidence of the solvated Li-ion co-intercalation reaction into graphite; however, detailed analysis was not provided. Yamada et al. further investigated the effect of the solvation nature of DMSO-based electrolytes on Li-ion co-intercalation into graphite (Yamada et al., 2010). By altering the salt concentrations and adding dimethyl carbonate (DMC) solvents, the solvation number of DMSO molecules (N_DMSO_) for Li ions could be controlled. For N_DMSO_ >≈3, Li ions were intercalated without full desolvation of the DMSO solvents (i.e., co-intercalation), whereas the conventional bare Li-ion intercalation reaction was observed for N_DMSO_ < 2.

Kim et al. successfully demonstrated that practical battery performance can be achieved from an electrochemical system exploiting the co-intercalation of Li, as shown in Figure 5C (Kim et al., 2016b). Moreover, similar results were obtained for K and Na electrochemical cells with a graphite electrode. All the electrochemical cells delivered specific capacities of ≈100 mAh g^−1^ regardless of the inserted alkali ion species. In addition, the structural evolution and staging behavior were similar for Li, Na, and K co-intercalations, implying that the interaction between alkali ions and the graphite host was not predominant and had been significantly weakened by the presence of the solvating molecules (Figure 5D). However, the insertion potentials of the solvated-ion intercalations were clearly distinguished for each case. Figure 5E shows that the insertion potential of the co-intercalation reactions increased from Li to Na and K. It was speculated that because the expanded interlayer distance reduces the electrostatic repulsion between negatively charged adjacent graphene layers, larger guest ions may stabilize the discharged GIC more, leading to a higher potential. Considering the diversity of solvation structures, 12-crown-4-ether solvents were also studied to understand the Li co-intercalation mechanism in graphite (Shimizu et al., 2018). A higher rate performance was realized by employing 12-crown-4-ether solvents in the co-intercalation rather than the conventional ethylene carbonate (EC)/DMC electrolyte system. Upon increasing the current density from 0.1 C to 5 C, 20% of the capacity was maintained with the EC/DMC electrolyte system, whereas 66% of the capacity was retained with the 12-crown-4-ether electrolyte system. This improvement was attributed to the absence of the desolvation process during the intercalation. However, the cycle stability was noted as a severe problem in Li co-intercalation reactions. Recently, Kim et al. showed that the main cause of the previously observed cycle degradation of Li co-intercalation was indeed related to side reactions at the surface of the Li metal counter electrode used in the half-cell experiment rather than the co-intercalation itself, and the side reactions arose from the chemical incompatibility between Li metal and DEGDME-based electrolytes (Kim et al., 2017). They demonstrated the stable cycling of LiFePO_4_/graphite full cells with ≈80% capacity retention after 200 cycles. However, Jung et al. claimed that Li co-intercalation provided relatively lower power capability compared with Na co-intercalation into graphite. DFT calculations indicated that the diglyme-solvated Li ions possess a curved solvated structure, leading to steric hindrance with other molecules during co-intercalation reactions, in contrast to the rapid diffusion of the flat Na-diglyme structure in graphite galleries (Jung et al., 2017).

The electrochemical behavior for co-intercalation differs from that of the conventional intercalation of Li or K ions into graphite, with the most practical and obvious dissimilarities being the average voltage and specific capacity. When the solvent intercalates together with Li or K ions in LIBs or K-ion batteries (KIBs), the GICs, the product of the co-intercalation, are likely to be energetically more stabilized as the solvent molecules alleviate the unfavorable interaction between the graphene layer and intercalated ions. The higher stability in the product of the electrochemical reaction leads to a higher discharge potential for the co-intercalation reaction. The voltages for co-intercalation are generally 0.5–1 V, which are much higher than the ≈0.2 V of the bare-ion intercalation. With respect to the specific capacity, the graphite electrode is capable of delivering a capacity of 372 and 250 mAh g^−1^ for bare Li and K insertion, respectively. However, for co-intercalation, because solvent molecules are involved in the intercalant weight and restrict the space for the occupation of alkali ions, a loss of capacity is inevitable; the capacity is typically reduced to ≈140 mAh g^−1^. Another important difference is that relatively faster charge/discharge kinetics has been generally observed for co-intercalation despite the bulky solvent–ion complex intercalation. One possible reason for this observation is that the desolvation process is not involved in the co-intercalation reactions. Because the desolvation step at the electrode interface is regarded as one of the rate-determining steps in the charge-transfer reaction in graphite, the absence or simplification of the desolvation process may contribute to enhanced rate performance. The fast co-intercalation process has also been attributed to the nature of the SEI, as limited growth of the SEI layer has typically been observed for the co-intercalation of Li, Na, and K. Kim et al. demonstrated that approximately 87% of the theoretical capacity of a graphite co-intercalation anode could be retained upon increasing the current density from 0.05 to 1 A g^−1^(Kim et al., 2017). Comparison of Li, K intercalation and co-intercalation is summarized in Table 1, which will provide useful guidance toward selection of intercalation processes for different end applications.

**Table 1:** Comparison of the co-intercalation and conventional intercalation of Li and K in graphite, illustrating the advantage and disadvantage for Li or K co-intercalation.

	**Co-intercalation**	**Conventional intercalation**
Average voltage	High (Bad)	Low
Specific capacity	Low (Bad)	High
Rate capability	High (Good)	Low
Volume change	Large (Bad)	Small
Diversity of Chemistry	High (Good)	Low

### Multi-Valent Cation Co-intercalation

**Figure 6:**
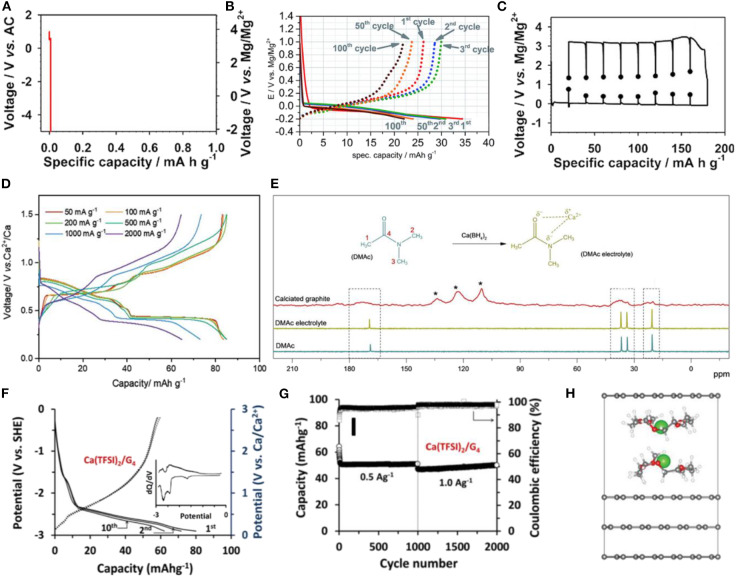
Charge and discharge profiles of graphite with **(A)** conventional carbonate-based Mg electrolyte and **(B)** DMF-based Mg electrolyte. **(C)** Galvanostatic intermittent titration technique (GITT) profiles of graphite with DME/DEGDME-based Mg electrolyte. **(D)** Charge and discharge profiles of graphite with DMAc-based Ca electrolyte at different current densities. **(E)** ^13^C-NMR spectra of DMAc solvent, DMAc-based electrolyte, and fully calciated graphite. **(F)** Charge and discharge profiles of graphite with TEGDME-based Ca electrolyte and the corresponding dQ/dV curve. **(G)** Cycle stability of graphite Ca cells with TEGDME-based electrolyte. **(H)** Energetically most probable configuration of Ca-TEGDME complex intercalated graphite. **(A,C)** Reproduced from Kim D. M. et al. (2018) with permission from American Chemical Society. **(B)** Reproduced from God et al. (2017) with permission from the Royal Society of Chemistry. **(D,E)** Reproduced from Park et al. (2019) with permission from Wiley-VCH. **(F–H)** Reproduced from Prabakar et al. (2019) with permission from Wiley-VCH. Note that three peaks at 109.8, 123, and 133.7 ppm indicated with ^*^ are originated from graphite and/or carbon peaks of binder.

Recently, the exploitation of multi-valent ions has been heavily investigated in the battery field. Rechargeable batteries based on multi-valent ions are expected to be one of the possible solutions for increasing the energy density by using double or triple the number of electrons per one ion intercalation, taking advantage of a similar intercalation host/reaction with doubled or more-than-doubled capacity (Aurbach et al., 2000; Lin et al., 2015; Ponrouch et al., 2016; Kim et al., 2019). In particular, Mg and Ca batteries have attracted widespread interest because of their low cost, light weight, and stable multi-valent states. Mg and Ca metals are the natural choice for an anode for such a battery system because the elemental metal can offer the highest energy density. Nevertheless, thick insulating films generally form on the surfaces of these elemental metal anodes when assembled in an electrochemical system employing common organic solvents, and these passivation layers typically inhibit the reversible deposition/stripping of Mg or Ca, leading to poor electrochemical performance (Gummow et al., 2018; Wang et al., 2018). As an alternative anode, graphite has been considered as an anode host for Mg and Ca, establishing MIBs or CIBs. However, similar to Na GICs, Mg or Ca binary GICs are difficult to synthesize or obtain electrochemically at ambient conditions (Xu and Lerner, 2018). Preparation of stage 1 GICs usually requires several weeks of heat treatments at 400°C−500°C, and the electrochemical formation is not feasible in conventional electrolyte systems (Figure 6A), which has hindered the development of graphite-based anodes for MIBs and CIBs.

Recently, God et al. claimed that a new electrolyte system may enable Mg co-intercalation into graphite (God et al., 2017). Figure 6B shows that a graphite cell with 0.5 M magnesium bis(trifluoromethanesulfonimide) (Mg(TFSI)_2_) in dimethylformamide (DMF) as the electrolyte reversibly delivers a capacity of ≈35 mAh g^−1^ at the first cycle and that this capacity is sustained for 100 cycles with a coulombic efficiency of ≈98%. Although *ex-situ* XRD analysis was performed to provide evidence of the possibility of solvent co-intercalation with Mg ions, the analysis did not clearly support the solvated-ion intercalation. Moreover, the co-intercalation voltage was abnormally low (<0 V vs. Mg/Mg^2+^), which contrasts with the values previous reported for other co-intercalation systems. More recently, Kim et al. used an ether-based electrolyte to enable a Mg co-intercalation reaction (Kim D. M. et al., 2018). By employing 0.3 M Mg(TFSI)_2_ in DME/DEGDME as the electrolyte, the cell could be galvanostatically cycled with a specific capacity of 180 at 2 mA g^−1^. A large polarization of ≈2 V was observed during the de/intercalation reactions, as shown in Figure 6C. *Ex-situ* XRD combined with FTIR spectroscopy revealed the presence of solvent molecules intercalated within the magnesiated graphite. Additionally, a Mg^2+^-DEGDME double-layer structure was proposed as the most stable configuration of fully discharged graphite according to DFT calculations. It is worth noting that the concept of improving Mg^2+^ intercalation kinetics through solvent chelation has been expended to MgCl^+^, which lowers the polarization strength and activation barrier for Mg^2+^, leading to high power Mg ion batteries with TiS_2_ (Yoo et al., 2017) or VOPO_4_ (Zhou et al., 2018) electrodes.

A more recent study also revealed that the room-temperature electrochemical insertion of Ca^2+^ in a graphite electrode is feasible utilizing the co-intercalation reaction (Park et al., 2019). Park et al. demonstrated that a graphite cell with 0.5 M Ca(BH_4_)_2_ in dimethylacetamide (DMAc) as the electrolyte exhibited a reversible capacity of 97 mAh g^−1^ at the first cycle, which was stably retained in subsequent cycles. Moreover, the graphite electrode delivered a capacity of 67 mAh g^−1^ at a high rate of 2.0 A g^−1^, corresponding to 75% of the capacity delivered at 0.05 A g^−1^ (Figure 6D), which is a respectable power capability. *In operando* XRD data suggested that an analogous staging process to that of Na co-intercalation occurred in the Ca electrochemical cell, confirming the co-intercalation. Additionally, a possible Ca^2+^-DMAc complex structure was proposed based on the results of solid-state nuclear magnetic resonance (NMR) and DFT calculation, as depicted in Figure 6E (Park et al., 2019). Prabakar et al reported that the use of 1 M Ca(TFSI)_2_ in tetraglyme as the electrolyte could also enable Ca^2+^-ion intercalation together with the ether solvent. The graphite electrode delivered a reversible capacity of ≈80 mAh g^−1^ at the first cycle (Figure 6F) (Prabakar et al., 2019). In addition, the capacity was maintained for more than 2,000 cycles at a current density of 1.0 A g^−1^, as shown in Figure 6G. According to DFT calculations, when tetraglyme is co-intercalated with calcium ions, a parallel double-stacking configuration is formed in the GICs, which is similar to the observation for the previous Na ether co-intercalation. Nevertheless, a stage 1 calciated graphite structure has not been reported in the literatures, and this structure is speculated to possess larger electrostatic interactions between Ca^2+^ ions than those of alkali metal ions; the stage 3 configuration is shown in Figure 6H. Further studies are needed to elucidate the specific solvent selectivity that makes multivalent-ion co-intercalation possible.

## Advantages and Challenges for Solvated-ion Intercalation

As observed in the survey of previous experimental and theoretical findings, there are several unique advantages of the co-intercalation reactions of graphite in an electrochemical system. First, the kinetics of solvated-ion intercalation is remarkably faster than that of conventional intercalation reactions. As demonstrated by Kim et al., the [Li–ether]^+^ co-intercalation into graphite can be performed at up to 1 A g^−1^ without a noticeable reduction in capacity; in contrast, the conventional Li intercalation capacity at 1 A g^−1^ is significantly smaller (Kim et al., 2017). Experimental and simulation studies have suggested that the lack of a desolvation process during the co-intercalation and the marginal formation of a SEI layer at the graphite surface synergistically promote the facile transport of solvated ions into graphite (Jung et al., 2017). The minimized interaction between the solvated guest ions and graphene layers also contributes to the rapid diffusion of the intercalant in graphite. For example, DMAc-solvated-Ca ions were readily intercalated into graphite (Park et al., 2019), delivering an exceptionally high rate capability up to 2 A g^−1^, with 75% of the specific capacity maintained at 0.05 A g^−1^. The fast intercalation kinetics are beneficial for achieving high-power-density full cells; for example, graphite//Na_1.5_VPO_4.8_F_0.7_ full cells possessed the highest-ever reported power density of 3,863 Wh kg^−1^ among Na-ion batteries (Xu et al., 2019). Second, the co-intercalation of the graphite anode generally results in remarkable cycle stability (up to several thousand cycles, as shown in Table 2), which is appealing for the practical application of rechargeable batteries. First-principles calculations revealed that Na–ether co-intercalated graphite exhibits robust stability owing to the ether–graphene van der Waals interaction (Jung et al., 2017; Yoon et al., 2017), ensuring notable cyclic stability for co-intercalation electrodes. Third, the thin and robust SEI is also believed to improve the cyclic reversibility and lead to high coulombic efficiency and fast reaction kinetics for the co-intercalation of graphite. Various characterization tools have revealed the presence of unusually thin and stable SEI layers at the graphite surface during co-intercalation reactions. XPS combined with depth profiling clearly confirmed that only a marginal amount of SEI was formed on the surface of graphite in LIBs using an ether-based electrolyte (Kim et al., 2017). Similarly, TEM analysis revealed a thin SEI for Na co-intercalated graphite (Goktas et al., 2018b). Kim et al. attributed the absence of or unusually thin surface film to the relatively high cut-off voltage (0.3 V *vs*. Li^+^/Li) of the Li co-intercalation and the high LUMO level of the Li–ether-based electrolyte (Kim et al., 2017). Goktas et al. assumed that the interface between graphite and the electrolyte is kinetically stabilized in the Na co-intercalation system, thereby suppressing the decomposition of the electrolyte (Goktas et al., 2018b). Understanding the unprecedentedly thin SEI at the graphite surface during co-intercalation is thus an important future task.

**Table 2:** Electrochemical performance of reported co-intercalation graphite anodes in rechargeable batteries.

**Batteries/electrodes**	**Cyclability/ mAh g^**−1**^**	**Rate capability/ mAh g^**−1**^**	**References**
LIB/graphite// LiFePO_4_	120 at 0.05A g^−1^ for the 1st cycle 96 at 0.05 A g^−1^ after 200 cycles	≈120 at 0.05 A g^−1^, 110 at 0.5 A g^−1^ and ≈100 at 1 A g^−1^	Kim et al., 2017
SIB/ graphite//Na metal	150 at 0.1 A g^−1^ for the 1st cycle 100 at 0.5 A g^−1^ after 2,500 cycles	150 at 0.1 A g^−1^, 100 at 5 A g^−1^, 75 at 10 A g^−1^	Kim et al., 2015a
SIB/ graphite// Na_1.5_VPO_4.8_F_0.7_	103 at 0.1 A g^−1^ for the 1st cycle ≈70 at 0.5 A g^−1^ after 250 cycles	103 at 0.1 A g^−1^, ≈40 at 1.0 A g^−1^	Kim et al., 2015a
SIB/ graphite// Na metal	≈100 at 37 mA g^−1^ for the 1st cycle, ≈87 at 37.2 mA g^−1^ after 100 cycles	≈100 at 37.2 mA g^−1^, ≈80 at 372 mA g^−1^	Jache and Adelhelm, 2014
SIB/graphene foam//Na metal	≈150 at 0.2 A g^−1^ for the 1st cycle ≈120 at 12 A g^−1^ after 8,000 cycles	≈150 at 1 A g^−1^, ≈125 at 10 A g^−1^ and ≈100 at 30 A g^−1^	Cohn et al., 2016b
SIB/ graphite// Na_1.5_VPO_4.8_F_0.7_	≈120 at 0.1 A g^−1^ for the 1st cycle ≈112 at 1 A g^−1^ after 1,000 cycles	≈120 at 0.1 A g^−1^, ≈110 at 1 A g^−1^, ≈100 at 4 A g^−1^	Xu et al., 2019
SIB/graphite//Na metal	110 at 0.1 A g^−1^ for the 1st cycle ≈110 at 0.2 A g^−1^ after 6,000 cycles	≈110 at 0.1 A g^−1^, ≈102 at 10 A g^−1^	Zhu et al., 2015
SIB/ graphite// Na_3_V_2_(PO_4_)_3_	≈90 at 0.1 A g^−1^ for the 1st cycle ≈75 at 2 A g^−1^ after 400 cycles	≈90 at 0.2 A g^−1^, ≈85 at 0.5 A g^−1^ and 80 at 10A g^−1^	Zhu et al., 2015
KIB/ graphite// Potassium Prussian Blue	≈46 at 0.2 A g^−1^ for the 1st cycle ≈30 at 2 A g^−1^ after 2,000 cycles	≈44 at 0.5 A g^−1^, ≈36 at 1.0 A g^−1^, ≈25 at 2.0 A g^−1^ and 15 at 3.0 at A g^−1^	Moyer et al., 2018
KIB/ graphite// K metal	87 at 0.28 A g^−1^ for the 1st cycle ≈73 at 2.8 A g^−1^ after 3,500 cycles	90 at 0.14 A g^−1^ and 82 at 2.8 A g^−1^	Wang et al., 2019
CIB/graphite//Ca metal	≈62 at 0.05 A g^−1^ for the 1st cycle ≈50 at 1 A g^−1^ after 2,000 cycles	≈80 at 0.05 A g^−1^, ≈55 at 0.5 A g^−1^ and ≈50 at 1 A g^−1^	Prabakar et al., 2019
CIB/graphite//Ca metal	≈87 at 0.05 A g^−1^ for the 1st cycle ≈83 at 0.1 A g^−1^ after 40 cycles	85 at 0.1 A g^−1^, 75 at 1 A g^−1^ and 65 at 2 A g^−1^	Park et al., 2019
MIB/ graphite//Mg metal	≈200 at 2 mA g^−1^ for the 1st cycle N/A	N/A	Kim D. M. et al., 2018

*Note that the capacity of full cell is based on the mass of an anode*.

Co-intercalation in graphite is not without issues, including (i) the large volume expansion of graphite during discharge, (ii) the high redox potential, (iii) the request of a flooded electrolyte, and (iv) the poor understanding of the reaction mechanisms. In commercial LIB configurations, the swelling of the external dimensions of batteries is generally limited to below 5% to guarantee the safety and stability of battery packs (Dash and Pannala, 2016). However, the co-intercalation of graphite typically involves large volume variations (200–300% for [Na–ether]^+^ (Kim et al., 2015b) and 215% for [Ca–DMAc]^2+^ (Park et al., 2019) co-intercalation, respectively). Recently, an ether–amine co-solvent electrolyte was reported to significantly reduce the volume change of graphite during discharge compared with the pure ether system (Zhang et al., 2018). However, the reported co-solvent system exhibited relatively low coulombic efficiency and poor cyclic stability, limiting its practical application. In addition, the co-intercalation redox potentials are comparatively high for anode application, i.e., 0.6–0.8 V vs. Na^+^/Na for SIBs (Xu et al., 2019), 0.75 V vs. Li^+^/Li for LIBs (Kim et al., 2017), and 0.6 V vs. Ca^2+^/Ca for CIBs (Park et al., 2019; Prabakar et al., 2019). These values are much higher than the 0.15 V *vs*. Li^+^/Li for the conventional intercalation reactions of a graphite anode in LIBs (Shu et al., 1993). Decreasing the co-intercalation potential to achieve high-voltage and high-energy full cells remains a challenge. Another factor that reduces the energy density of graphite-based full cells is the participation of the electrolyte in the active co-intercalation reactions. Using graphite//Na_1.5_VPO_4.8_F_0.7_ in 2 M DME NaPF_6_ electrolyte as an example, the practical energy density is estimated to be only 23.8 Wh kg^−1^ based on the amount of electrolyte used in the co-intercalation reaction (Xu et al., 2019). This value is far from the theoretical value of 149 Wh kg^−1^ based on the total mass of the electrodes. To approach the high theoretical energy densities, more efforts are needed to decrease the electrolyte/electrode ratio. In addition to the above practical concerns, there are many issues related to the fundamentals of co-intercalation reactions. For example, only linear ether-based electrolytes have been confirmed to be capable of Na, Li, K, and Mg co-intercalation into graphite, and the Ca co-intercalation reaction has only been reported in DMAc-based electrolyte. Yoon et al. attempted to explain the solvent-selective Na co-intercalation from the viewpoint of the chelate effect and the stability of the co-intercalant in graphite (Yoon et al., 2017). More intensive studies are needed to comprehensively explain the solvent-selective property for co-intercalation and to screen for optimal solvents with universal co-intercalation capability and wide potential windows. In addition, Kim et al. proposed a standard staging process for [Na–ether]^+^ co-intercalation in graphite (Kim et al., 2015b), where [Na–ether]^+^ was inserted in every third layer for stage 3 GICs and every second layer for stage 2 GICs, based on *in-situ* synchrotron XRD results. However, the classic staging process cannot explain the phase transition from stage 3 to stage 2 Na-GICs. The Daumas–Herold staging mechanism proposed by Seidl et al. may more plausibly explain the above phase transition via continuous lattice filling with [Na–ether]^+^ complexes (Seidl et al., 2017). A clear understanding of the phase change and reaction kinetics for co-intercalation remains elusive.

## Summary and Perspectives

Graphite can serve as a promising host to accommodate a wide range of species, including alkali and alkaline earth elements, owing to its layered structure and unique amphoteric redox character. For the past three decades, graphite has been widely used as a standard anode material in LIBs, where Li ions are reversibly inserted/extracted into/from graphite galleries, delivering a high capacity. However, until recently, Na^+^, Mg^2+^, and Ca^2+^ ions could not be intercalated into graphite because of the strong local interactions between graphene and Na^+^, Mg^2+^, and Ca^2+^. Solvent–ion co-intercalation reactions successfully overcame these challenges were beneficial to the realization of substantial achievements in non-Li-ion rechargeable batteries. Specifically, ether-solvated Na ions were inserted into graphite with remarkable cyclic stability and power capability, reversible Ca-ion insertion into graphite was possible in a room-temperature electrochemical system with the co-intercalating DMAc solvent, and Mg ions were electrochemically inserted into graphite using ether electrolytes, delivering respectable energy and power density. Although it is not trivial to clarify the conditions for co-intercalation reactions, simulation studies have indicated that a strong solvation energy of the [solvent–ion] complexes and high LUMO levels of the intercalant are prerequisites for the co-intercalation reactions. For a long cycling test of rechargeable batteries with co-intercalation reactions, the stability of the counter electrode is important, and the interpretation of the cycle stability should not be misleading. The reaction dynamics and GIC configurations vary for co-intercalation reactions in different battery systems. In fully calciated graphite, one Ca ion is coordinated with four DMAc molecules during intercalation, whereas only one ether molecule chelates with one Na ion in fully sodiated graphite. The key to understanding the above differences lies in the fundamentals related to the solvated-ion configuration, charge-transfer mechanisms, and electrolyte systems, which should be emphasized in future studies.

On the basis of the progress and potential challenges for the co-intercalation of graphite summarized in this review, we propose the following suggestions for the future development of co-intercalation graphite anodes in rechargeable batteries.

### Exploration of Reliable Electrolytes

Electrolytes are the key component that can be used to enable/tune co-intercalation reactions and dramatically affect the electrochemical properties. As discussed in section Na Co-Intercalation and Li and K Co-Intercalation, ether-based electrolytes have been widely used in Li-, K-, and Na-ion co-intercalation reactions. However, compared with conventional carbonate-based electrolyte for intercalation reactions, ether-based electrolytes tend to exhibit low oxidation stability (i.e., ≈4 V for lithium bis(trifluoromethanesulfonyl)imide (LiTFSI) in DME vs. ≈5 V for LiTFSI in EC/PC) (Jiao et al., 2018) and low boiling points (i.e., 84°C for DME solvent vs. 250°C for EC/PC solvent) (Xu et al., 2018a). The former hinders the utilization of high-voltage cathodes for high-energy full batteries, and the latter leads to safety issues for practical applications. As discussed in section Multi-Valent Cation Co-Intercalation, the same problem has also been observed in DMAc-solvated-Ca co-intercalation reactions. The oxidation stability of the Ca(BH_4_)_2_ DMAc electrolyte is limited by approximately 2.9 V vs. Ca^2+^/Ca (Park et al., 2019), which significantly complicates the demonstration of Ca-ion full batteries with graphite anodes and transition metal oxide cathodes [i.e., 3.4 V *vs*. Ca^2+^/Ca for Na_x_MnFe(CN)_6_ (Lipson et al., 2015) and 3.5 V *vs*. Ca^2+^/Ca for CaCo_2_O_4_ (Cabello et al., 2016)]. With respect to the co-intercalation of solvated ions into graphite, the redox potentials are generally high, eventually resulting in unsatisfactory energy densities. Overall, when considering both the fundamental and practical challenges for electrolyte systems for co-intercalation reactions, the battery community can further improve the performance of batteries by investigating the components and functional mechanisms of the electrolytes.

### Decrease of Swelling of Graphite

The swelling of graphite during co-intercalation reactions is far from satisfactory to meet the standards for practical rechargeable batteries. The volume expansion of commercial LIBs at the pack level should typically be no more than 5% to ensure safety and cyclic performance. In the development of new battery systems, the improvement in energy density and/or reduction in cost is derived with the assumption that external dimensional change of the anode is constrained. However, the theoretical volume expansion of fully co-intercalated graphite is close to 200% (see section Advantages and Challenges for Solvated-Ion Intercalation). Graphite electrodes were reported to periodically expand/contract by 70%−100% during cycling because of cavities in the electrodes, the gap between the electrode and separator, and the cushion from the separator, in an *in-situ* electrochemical dilatometry study (Goktas et al., 2018b). Decreasing the swelling of graphite electrodes during co-intercalation without limiting the capacities, cycle life, and power capability remains an open challenge. Several potential strategies are suggested: (i) tailoring the morphology of graphite anodes, i.e., using graphite foam electrodes (note that excessive porosity may decrease the volumetric energy density of batteries), (ii) rationally limiting the co-intercalation capacity, (iii) designing new electrolytes for minimum volume expansion during the co-intercalation, and (iv) devising new cell configurations that can compensate for the volume expansion of batteries.

### Development of Full Batteries With Practically High Energy Densities

Among reported co-intercalation graphite anodes, full cell performance has only been reported for LIBs and SIBs, signifying the infancy stage of the study and development of full batteries. There are three critical challenges for the development of full batteries with practically high energy densities, namely, (i) the high redox potential and low specific storage capacity of graphite electrodes, (ii) the required minimum amount of electrolyte, and (iii) the unsuitable electrolytes for cathodes. During intercalation of Li ions in graphite in an EC/PC-based electrolyte, the operation potential and reversible capacity were ≈0.15 V vs. Li^+^/Li and 360 mAh g^−1^, respectively, whereas these values were ≈0.75 V vs. Li^+^/Li and 120 mAh g^−1^ for co-intercalation reactions in a DEGDME-based electrolyte, respectively (Kim et al., 2017). As a result, the energy densities of graphite//LiFePO_4_ full cells in the DEGDME- and EC/PC-based electrolytes were calculated to be approximately 162 and 297 Wh kg^−1^, respectively, based on the mass of electrode materials. A similar challenge is also faced for SIBs. The energy density of graphite//Na_1.5_VPO_4.8_F_0.7_ full cells in a 2 M DME-based electrolyte was determined to be approximately 112 Wh kg^−1^ (based on the total mass of electrode materials) (Xu et al., 2019); however, this value is lower than that of the cell of hard carbon//Na_0.9_[Cu_0.22_Fe_0.3_Mn_0.48_]O_2_ (210 Wh kg^−1^) (Mu et al., 2015). Although the long cycle life and high power capability for fast charge/discharge are appealing characteristics for co-intercalation reactions of graphite electrodes, improving the specific capacities and lowering the redox potential are important directions for future studies. As mentioned in section Solvated Alkali and Alkaline Earth Metal-Ion Intercalation in Graphite, the co-intercalation reactions involve the consumption of the electrolyte in the electrode reaction. To ensure reversible co-intercalation reactions, the inclusion of extra electrolyte is necessary in graphite-based cells, which would significantly lower the practical energy density of full batteries. For example, when the flooded amount of electrolyte (100 μl per cell) is considered, the energy density of graphite//Na_1.5_VPO_4.8_F_0.7_ full cells in 2 M DME-based electrolyte is only approximately 23.8 Wh kg^−1^ (*vs*. 146 Wh kg^−1^ based on the total mass of electrode materials and minimum amount of electrolyte) (Xu et al., 2019). To achieve high energy densities for full batteries, high-voltage cathodes must be employed. However, increasing the cut-off voltage for graphite//Na_1.5_VPO_4.8_F_0.7_ in DEGDME-based electrolyte was shown to be unfavorable for cyclic stability (i.e., 70% capacity retention after 200 cycles between 0.7 and 4.2 V (Kim et al., 2015a) vs. 92% capacity retention after 200 cycles between 0.7 and 3.8 V (Xu et al., 2019). In addition to the scientific challenges related to electrolyte systems, the safety, manufacturing cost, and feasibility of electrode materials should also be considered for further development of graphite anodes using the new co-intercalation reactions.

Based on abovementioned perspectives, we anticipate that novel design of graphite host materials using solvated ion intercalation chemistry will provide an unexplored pathway toward the realization of high-power and long-lasting post LIBs.

## Author Contributions

JP and Z-LX contributed equally to this work. KK supervised all aspects of the work.

## Conflict of Interest

The authors declare that this study received funding from Shell International Exploration & Production, Inc. The funder was not involved in the study design, collection, analysis, interpretation of data, the writing of this article or the decision to submit it for publication. The authors declare that the research was conducted in the absence of any commercial or financial relationships that could be construed as a potential conflict of interest.

## References

[B1] Abe T. Fukuda H. Iriyama Y. Ogumi Z. (2004). Solvated Li-ion transfer at interface between graphite and electrolyte. J. Electrochem. Soc. 151, 1120–1123. 10.1149/1.1763141

[B2] Abe T. Mizutani Y. Kawabata N. Inaba M. Ogumi Z. (2002). Effect of co-intercalated organic solvents in graphite on electrochemical Li intercalation. Synth. Met. 125, 249–253. 10.1016/S0379-6779(01)00538-0

[B3] Adhoum N. Bouteillon J. Dumas D. Poignet C. J. (2006). Electrochemical insertion of sodium into graphite in molten sodium fluoride at 1025°C. Electrochi. Acta 51, 5402–5406. 10.1016/j.electacta.2006.02.019

[B4] Armand M. Tarascon J. M. (2008). Building better batteries. Nature 451, 652–657. 10.1038/451652a 18256660

[B5] Aurbach D. Lu Z. Schechter A. Gofer Y. Gizbar H. Turgeman R.. (2000). Prototype systems for rechargeable magnesium batteries. Nature 407, 10–14. 10.1038/35037553 11048714

[B6] Besenhard J. O. Mohwald H. Nickl J. J. (1980). Electronic conductivity and structure of DMSO-solvated A^+^ and ${\text{NR}}_{4}^{+}$-graphite intercalation compounds. Carbon 18, 399–405. 10.1016/0008-6223(80)90031-7

[B7] Cabello M. Nacimiento F. González J. R. Ortiz G. Alcántara R. Lavela P. (2016). Advancing towards a veritable calcium-ion battery: CaCo_2_O_4_ positive electrode material. Electrochem. Commun. 67, 59–64. 10.1016/j.elecom.2016.03.016

[B8] Choi J. W. Aurbach D. (2016). Promise and reality of post-lithium-ion batteries with high energy densities. Nat. Rev. Mater. 1, 1–16. 10.1038/natrevmats.2016.13

[B9] Chung G. C. Kim H. J. Yu S. I. Jun S. H. Choi J. W. Kim M. H. (2000). Origin of graphite exfoliation-an investigration of the important role of solvent cointercalation. J. Electrochem. Soc. 147, 4391–4398. 10.1149/1.1394076/meta

[B10] Cohn A. P. Muralidharan N. Carter R. Share K. Oakes L. Pint C. L. (2016a). Durable potassium ion battery electrodes from high-rate cointercalation into graphitic carbons. J. Mater. Chem. A 4, 14954–14959. 10.1039/c6ta06797b

[B11] Cohn A. P. Share K. Carter R. Oakes L. Pint C. L. (2016b). Ultrafast solvent-assisted sodium ion intercalation into highly crystalline few-layered graphene. Nano Lett. 16, 543–548. 10.1021/acs.nanolett.5b04187 26618985

[B12] Dahn J. R. Zheng T. Liu Y. Xue J. S. (1995). Mechanisms for lithium insertion in carbonaceous materials. Science 270, 590–593. 10.1126/science.270.5236.590

[B13] Dash R. Pannala S. (2016). The potential of silicon anode based lithium ion batteries. Mater. Today, 19, 483–484. 10.1016/j.mattod.2016.07.005

[B14] Doeff M. M. Ma Y. Visco S. J. de Jonghe L. C. (1993). Electrochemical insertion of sodium into carbon. J. Electrochem. Soc. 140, 169–170. 10.1149/1.2221153

[B15] Dresselhaus M. S. Dresselhaus G. (1981). Advances in physics intercalation compounds of graphite. Advan. Phys. 30, 139–326. 10.1080/00018738100101367

[B16] Dunn B. Kamath H. Tarascon J. M. (2011). Electrical energy storage for the grid: a battery of choices. Science 334, 928–935. 10.1126/science.1212741 22096188

[B17] Fong R. Sacken U. van Dahn J. R. (1990). Studies of lithium intercalation into carbons using nonaqueous electrochemical cells. J. Electrochem. Soc. 137, 2009–2013. 10.1149/1.2086855/meta

[B18] Ge P. Fouletier M. (1988). Electrochemical intercalation of sodium in graphite. Solid State Ion. 28, 1172–1175. 10.1016/0167-2738(88)90351-7

[B19] God C. Bitschnau B. Kapper K. Lenardt C. Schmuck M. Mautner F. (2017). Intercalation behaviour of magnesium into natural graphite using organic electrolyte systems. RSC Adv. 7, 14168–14175. 10.1039/c6ra28300d

[B20] Goktas M. Akduman B. Huang P. Balducci A. Adelhelm P. (2018a). Temperature-induced activation of graphite co-intercalation reactions for glymes and crown ethers in sodium-ion batteries. J. Phys. Chem. C 122, 26816–26824. 10.1021/acs.jpcc.8b07915

[B21] Goktas M. Bolli C. Berg E. J. Novák P. Pollok K. Langenhorst F. (2018b). Graphite as cointercalation electrode for sodium-ion batteries: electrode dynamics and the missing solid electrolyte interphase (SEI). Adv. Energy Mater. 17:1702724 10.1002/aenm.201702724

[B22] Gummow R. J. Vamvounis G. Kannan M. B. He Y. (2018). Calcium-ion batteries: current state-of-the-art and future perspectives. Adv. Mater. 30:1801702. 10.1002/adma.201801702 29984434

[B23] Hasa I. Dou X. Buchholz D. Shao-Horn Y. Hassoun J. Passerini S. (2016). A sodium-ion battery exploiting layered oxide cathode, graphite anode and glyme-based electrolyte. J. Power Sources 310, 26–31. 10.1016/j.jpowsour.2016.01.082

[B24] Henderson W. A. (2006). Glyme-lithium salt phase behavior. J. Phys. Chem. B 110, 13177–13183. 10.1021/jp061516t 16805630

[B25] Irisarri E. Ponrouch A. Palacin M. R. (2015). Review - Hard carbon negative electrode materials for sodium-ion batteries. J. Electrochem. Soc. 162, A2476–A2482. 10.1149/2.0091514jes

[B26] Jache B. Adelhelm P. (2014). Use of graphite as a highly reversible electrode with superior cycle life for sodium-ion batteries by making use of co-intercalation phenomena. Angew. Chem. Int. Ed. 53, 10169–10173. 10.1002/anie.201403734 25056756

[B27] Jache B. Binder J. O. Abe T. Adelhelm P. (2016). A comparative study on the impact of different glymes and their derivatives as electrolyte solvents for graphite co-intercalation electrodes in lithium-ion and sodium-ion batteries. Phys. Chem. Chem. Phys. 18, 14299–14316. 10.1039/C6CP00651E 27165175

[B28] Jiao S. Ren X. Cao R. Engelhard M. H. Liu Y. Hu D. (2018). Stable cycling of high-voltage lithium metal batteries in ether electrolytes. Nat. Energy 3, 739–746. 10.1038/s41560-018-0199-8

[B29] Jow R. Delp S. A. Allen J. L. Jones J-P. Smart M. C. (2018). Factors limiting Li^+^ charge transfer kinetics in Li-ion batteries. J. Electrochem. Soc. 165, A361–A367. 10.1149/2.1221802jes

[B30] Jung S. C. Kang Y. J. Han Y. K. (2017). Origin of excellent rate and cycle performance of Na^+^-solvent cointercalated graphite vs. poor performance of Li+-solvent case. Nano Energy 34, 456–462. 10.1016/j.nanoen.2017.03.015

[B31] Kang K. Meng Y. S. Breger J. Gery C. P. Ceder G. (2006). Electrodes with high power and high capacity for rechargeable lithium batteries. Science 311, 977–980. 10.1126/science.1122152 16484487

[B32] Kim D. J. Yoo D. Otley M. T. Prokofjevs A. Pezzato C. Owczarek M. (2019). Rechargeable aluminium organic batteries. Nat. Energy 4, 51–59. 10.1038/s41560-018-0291-0

[B33] Kim D. M. Jung S. C. Ha S. Kim Y. Park Y. Ryu J. H. (2018). Cointercalation of Mg^2+^ ions into graphite for magnesium-ion batteries. Chem. Mater. 30, 3199–3203. 10.1021/acs.chemmater.8b00288

[B34] Kim H. Hong J. Park Y. U. Kim J. Hwang I. Kang K. (2015a). Sodium storage behavior in natural graphite using ether-based electrolyte systems. Adv. Funct. Mater. 25, 534–541. 10.1002/adfm.201402984

[B35] Kim H. Hong J. Yoon G. Kim H. Park K. Y. Park M. S. (2015b). Sodium intercalation chemistry in graphite. Energy Environ. Sci. 8, 2963–2969. 10.1039/C5EE02051D

[B36] Kim H. Kim H. Ding Z. Lee M. H. Lim K. Yoon G. (2016a). Recent progress in electrode materials for sodium-ion batteries. Adv. Energy Mater. 6:1600943 10.1002/aenm.201600943

[B37] Kim H. Lim K. Yoon G. Park J. H. Ku K. Lim H. D. (2017). Exploiting lithium-ether co-intercalation in graphite for high-power lithium-ion batteries. Adv. Energy Mater. 7:1700418 10.1002/aenm.201700418

[B38] Kim H. Yoon G. Lim K. Kang K. (2016b). A comparative study of graphite electrodes using the co-intercalation phenomenon for rechargeable Li, Na and K batteries. Chem. Commun. 52, 12618–12621. 10.1039/C6CC05362A 27709171

[B39] Kim J. Kim H. Kang K. (2018). Conversion-based cathode materials for rechargeable sodium batteries. Adv. Energy Mater. 8:1702646 10.1002/aenm.201702646

[B40] Kim J.-H. Kim D. K. (2018). Conversion-alloying anode materials for Na-ion batteries: recent progress, challenges, and perspective for the future. J. Korean Ceram. Soc. 55, 307–324. 10.4191/kcers.2018.55.4.07

[B41] Kim S. W. Seo D. H. Ma X. Ceder G. Kang K. (2012). Electrode materials for rechargeable sodium-ion batteries: potential alternatives to current lithium-ion batteries. Adv. Energy Mater. 2, 710–721. 10.1002/aenm.201200026

[B42] Laziz N. A. Abou-Rjeily J. Darwiche A. Toufaily J. Outzourhit A. Ghamouss F. (2018). Li- and Na-ion storage performance of natural graphite via simple flotation process. J. Electrochem. Sci. Technol. 9, 320–329. 10.5229/JECST.2018.9.4.320

[B43] Lee S. Kwon G. Ku K. Yoon K. Jung S. K. Lim H. D. (2018). Recent progress in organic electrodes for Li and Na rechargeable batteries. Adv. Mater. 3:1704682 10.1002/adma.201704682 29582467

[B44] Li Y. Lu Y. Adelhelm P. Titirici M. M. Hu Y. S. (2019). Intercalation chemistry of graphite: alkali metal ions and beyond. Chem. Soc. Rev. 48, 4655–4687. 10.1039/c9cs00162j 31294739

[B45] Lin M. C. Gong M. Lu B. Wu Y. Wang D. Y. Guan M.. (2015). An ultrafast rechargeable aluminium-ion battery. Nature 520, 324–328. 10.1038/nature14340 25849777

[B46] Lipson A. L. Pan B. Lapidus S. H. Liao C. Vaughey J. T. Ingram B. J. (2015). Rechargeable Ca-ion batteries: a new energy storage system. Chem. Mater. 27, 8442–8447. 10.1021/acs.chemmater.5b04027

[B47] Liu Y. Merinov B. V. Goddard W. A. (2016). Origin of low sodium capacity in graphite and generally weak substrate binding of Na and Mg among alkali and alkaline earth metals. Proc. Natl. Acad. Sci. U.S.A. 113, 3735–3739. 10.1073/pnas.1602473113 27001855 PMC4833228

[B48] Matsui T. Takeyama K. (1998). Li^+^ adsorption on a metal electrode from glymes. Electrochim. Acta 43, 1355–1360. 10.1016/S0013-4686(97)10043-3

[B49] Metrot A. Guerard D. Billaud D. Herold A. (1979). New results about the sodium-graphite system. Synth. Met. 1, 363–369. 10.1016/0379-6779(80)90071-5

[B50] Moriwake H. Kuwabara A. Fisher C. A. J. Ikuhara Y. (2017). Why is sodium-intercalated graphite unstable? RSC Adv. 7, 36550–36554. 10.1039/C7RA06777A

[B51] Moyer K. Donohue J. Ramanna N. Cohn A. P. Muralidharan N. Eavesb J.. (2018). High-rate potassium ion and sodium ion batteries by co-intercalation anodes and open framework cathodes. Nanoscale 10, 13335–13342. 10.1039/c8nr01685b 29989632

[B52] Mu L. Xu S. Li Y. Hu Y. S. Li H. Chen L.. (2015). Prototype sodium-ion batteries using an air-stable and Co/Ni-free O3-layered metal oxide cathode. Adv. Mater. 27, 6928–6933. 10.1002/adma.201502449 26436288

[B53] Muldoon J. Bucur C. B. Gregory T. (2014). Quest for nonaqueous multivalent secondary batteries: magnesium and beyond. Chem. Rev. 114, 11683–11720. 10.1021/cr500049y 25343313

[B54] Nobuhara K. Nakayama H. Nakanishi S. Iba H. (2013). First-principles study on alkali metal-graphite intercalation compounds. J. Power Sources 243, 585–587. 10.1016/j.jpowsour.2013.06.057

[B55] Okamoto Y. (2014). Density functional theory calculations of alkali metal (Li, Na, and K) graphite intercalation compounds. J. Phys. Chem. C 118, 16–19. 10.1021/jp4063753

[B56] Olivetti E. A. Ceder G. Gaustad G. G. Fu X. (2017). Lithiumm ion battery supply chain considerations: analysis of potential bottlenecks in critical metals. Joule 1, 229–243. 10.1016/j.joule.2017.08.019

[B57] Pan H. Hu Y. S. Chen L. (2013). Room-temperature stationary sodium-ion batteries for large-scale electric energy storage. Energy Environ. Sci. 6:2338 10.1039/c3ee40847g

[B58] Park J. Xu Z. L. Yoon G. Park S. K. Wang J. Hyun H.. (2019). Stable and high-power calcium-ion batteries enabled by calcium intercalation into graphite. Adv. Mater. 32:1904411. 10.1002/adma.201904411 31736158

[B59] Ponrouch A. Frontera C. Bardé F. Palacín M. R. (2016). Towards a calcium-based rechargeable battery. Nat. Mat. 15, 169–172. 10.1038/nmat4462 26501412

[B60] Ponrouch A. Palacin M. R. (2018). On the road toward calcium-based batteries. Cur. Opin. Electrochem. 9, 1–7. 10.1016/j.coelec.2018.02.001

[B61] Prabakar S. J. R. Ikhe A. B. Park W. B. Chung K. Park H. Kim K. (2019). Graphite as a long-life Ca^2+^-intercalation anode and its implementation for rocking-chair type calcium-ion batteries. Adv. Sci. 16:1902129 10.1002/advs.201902129 PMC6918123 31890464

[B62] Rhodes C. P. Khan M. Frech R. (2002). Crystalline phases of poly(ethylene oxide)oligomers and sodium triflate: changes in coordination and conformation with chain length. J. Phys. Chem. B 106, 10330–10337. 10.1021/jp0141981

[B63] Schafhaeutl C. (1840). Ueber die verbindungen des kohlenstoffes mit silicium, Eisen und anderen Metallen, welche die verschiedenen Gallungen von Roheisen, Stahl und Schmiedeeisen bilden. J. Parkt. Chem. 21, 129–157. 10.1002/prac.18400210117

[B64] Seidl L. Bucher N. Chu E. Hartung S. Martens S. Schneider O. (2017). Intercalation of solvated Na-ions into graphite. Energy Environ. Sci. 10, 1631–1642. 10.1039/C7EE00546F

[B65] Shimizu M. Koya T. Umeki M. Arai S. (2018). Communication — Intercalation/de-intercalation behavior of Li-ion encapsulated by 12-Crown-4-ether into graphite electrode. J. Electrochem. Soc. 165, 3212–3214. 10.1149/2.0021814jes

[B66] Shu Z. X. Mcmillan R. S. Murray J. J. (1993). Electrochemical intercalation of lithium into graphite. J. Electrochem. Soc. 140, 922–927. 10.1149/1.2056228/meta

[B67] Stevens D. A. Dahn J. R. (2001). The mechanisms of lithium and sodium insertion in carbon materials. J. Electrochem. Soc. 148, A803–A811. 10.1149/1.1379565

[B68] Takeuchi S. Miyazaki K. Sagane F. Fukutsuka T. Jeong S. Abe T. (2011). Electrochemical properties of graphite electrode in propylene carbonate-based electrolytes containing lithium and calcium ions. Electrochim. Acta 56, 10450–10453. 10.1016/j.electacta.2011.06.062

[B69] Wagner M. R. Albering J. H. Moeller K. C. Besenhard J. O. Winter M. (2005). XRD evidence for the electrochemical formation of Li^+^(PC)_y_C_n_- in PC-based electrolytes. Electrochem. Commun. 7, 947–952. 10.1016/j.elecom.2005.06.009

[B70] Wang D. Gao X. Chen Y. Jin L. Kuss C. Bruce P. G. (2018). Plating and stripping calcium in an organic electrolyte. Nat. Mater. 17, 16–20. 10.1038/NMAT5036 29180779

[B71] Wang L. Yang J. Li J. Chen T. Chen S. Wu Z. (2019). Graphite as a potassium ion battery anode in carbonate-based electrolyte and ether-based electrolyte. J. Power Sources 409, 24–30. 10.1016/j.jpowsour.2018.10.092

[B72] Wang Z. Selbach S. M. Grande T. (2014). Van der Waals density functional study of the energetics of alkali metal intercalation in graphite. RSC Adv. 4, 4069–4079. 10.1039/C3RA47187J

[B73] Xiang X. Zhang K. Chen J. (2015). Recent advances and prospects of cathode materials for sodium-ion batteries. Adv. Mater. 27, 5343–5364. 10.1002/adma.201501527 26275211

[B74] Xu W. Lerner M. M. (2018). A new and facile route using electride solutions to intercalate alkaline earth ions into graphite. Chem. Mater. 30, 6930–6935. 10.1021/acs.chemmater.8b03421

[B75] Xu Z. L. Lim K. Park K. Y. Yoon G. Seong W. M. Kang K. (2018a). Engineering solid electrolyte interphase for pseudocapacitive anatase TiO_2_ anodes in sodium-ion batteries. Adv. Funct. Mater. 28:1802099 10.1002/adfm.201802099

[B76] Xu Z. L. Park J. Yoon G. Kim H. Kang K. (2018b). Graphitic carbon materials for advanced sodium-ion batteries. Small Methods 8:1800227 10.1002/smtd.201800227

[B77] Xu Z. L. Yoon G. Park K.-Y. Park H. Tamwattana O. Kim S. J.. (2019). Tailoring sodium intercalation in graphite for high energy and power sodium ion batteries. Nat. Commun. 10:2598. 10.1038/s41467-019-10551-z 31197187 PMC6565630

[B78] Yamada Y. Takazawa Y. Miyazaki K. Abe T. (2010). Electrochemical lithium intercalation into graphite in dimethyl sulfoxide-based electrolytes: effect of solvation structure of lithium ion. J. Phys. Chem. C 114, 11680–11685. 10.1021/jp1037427

[B79] Yoo H. D. Liang Y. Dong H. Lin J. Wang H. Liu Y.. (2017). Fast kinetics of magnesium monochloride cations in interlayer-expanded titanium disulfide for magnesium rechargeable batteries. Nat. Commun. 8:339. 10.1038/s41467-017-00431-9 28835681 PMC5569106

[B80] Yoon G. Kim H. Park I. Kang K. (2017). Conditions for reversible Na intercalation in graphite: theoretical studies on the interplay among guest ions, solvent, and graphite host. Adv. Energy Mater. 7:1601519 10.1002/aenm.201601519

[B81] Yun Y. S. Park K. Lee B. Cho S. Y. Park Y. Hong S. J.. (2015). Sodium-ion storage in pyroprotein-based carbon nanoplates. Adv. Mater. 27, 6914–6921. 10.1002/adma.201502303 26421382

[B82] Zhang H. Li Z. Xu W. Chen Y. Ji X. (2018). Pillared graphite anodes for reversible sodiation. Nanotechnology 29:325402. 10.1088/1361-6528/aac69a/meta 29785969

[B83] Zhou L. Liu Q. Zhang Z. Zhang K. Xiong F. Tan S.. (2018). Interlayer-spacing-regulated VOPO_4_ nanosheets with fast kinetics for high-capacity and durable rechargeable magnesium batteries. Adv. Mater. 30:1801984. 10.1002/adma.201801984 29939435

[B84] Zhu Z. Cheng F. Hu Z. Niu Z. Chen J. (2015). Highly stable and ultrafast electrode reaction of graphite for sodium ion batteries. J. Power Sources, 293, 626–634. 10.1016/j.jpowsour.2015.05.116

